# Internet Use by Parents of Children With Rare Conditions: Findings From a Study on Parents’ Web Information Needs

**DOI:** 10.2196/jmir.5834

**Published:** 2017-02-28

**Authors:** Honor Nicholl, Catherine Tracey, Thelma Begley, Carole King, Aileen M Lynch

**Affiliations:** ^1^ School of Nursing and Midwifery Trinity College Dublin, the University of Dublin Dublin Ireland

**Keywords:** rare diseases, parents, Internet, consumer health information, social media, professional-patient relations

## Abstract

**Background:**

Parents of children with rare conditions increasingly use the Internet to source information on their child’s condition. This study reports on part of a larger study whose overall aim was to identify the Internet use by parents when seeking information on their child’s rare condition, with the specific purpose of using the findings to aid in the development of a website specifically designed to meet the parents’ needs. It presents findings on why these parents use the Internet, the information and support content they source, and the impact these resources have on their capacity to care for and manage their child’s condition.

**Objective:**

To (1) ascertain parents’ general Internet usage patterns, (2) identify the nature of the information parents most frequently searched for, and (3) determine the effect the Internet-sourced information had on parents of children with rare conditions.

**Methods:**

Data collection was conducted in 2 parts: Part 1 was a focus group interview (n=8) to inform the development of the questionnaire, and Part 2 was a questionnaire (Web- and paper-based). All respondents (N=128) completed the questionnaire using the Internet.

**Results:**

Parents frequently and habitually used the Internet and social media to gather information on their child’s condition. These Web-based resources provide parents with a parent-to-parent support platform that allows them to share their experiences and information with other parents, which, the respondents considered, improved their knowledge and understanding of their child’s condition. The respondents also reported that these resources positively impacted on their decision making, care, and management of their child’s condition. However, they reported receiving mixed responses when wishing to engage and share with health care professionals their Internet and social media interactions and information outcomes.

**Conclusions:**

This study adds to the emerging body of research on the Internet use by parents of children with rare conditions to source information on their child’s condition. The evolving and ever increasing parent-to-parent support systems via social media are impacting on parents’ capacity to manage their children. Implications for practice include health care professionals’ response to this knowledge and capacity shift, and the significance of these changes when interacting with parents. The key message of this study was that parents of children with rare conditions are habitual users of the Internet to source information about their children’s conditions. Social media, especially Facebook, has an increasing role in the lives of these parents for information and support. Parents’ interest in information gathering and sharing includes a desire for shared dialogue with health care professionals.

## Introduction

The Internet is recognized as a significant source of health information [1] and may assist parents of children with rare conditions (*rare conditions* includes rare diseases and rare disorders in this paper) to find information, clarify, and understand the information they have been given, or confirm a diagnosis [2,3]. Parents of these children can experience difficulties with aspects of their child’s diagnosis and management, as each condition is rare and diverse in symptoms. Traditionally, health care professionals have been the gatekeepers of specialized knowledge about the child’s rare condition [4]. In some cases, the rareness of the child’s condition means that information is difficult to find, even for health care professionals, thus making diagnosis and management of the child and his or her condition difficult [4,5].

The Internet is changing the way parents of children with rare conditions access information. It enables them access worldwide information that previously was unavailable to them [6,7]. In comparison to more traditional sources, the Internet may provide large volumes of information from a variety of sources and perspectives, for example, emerging treatment options or relevant clinical trials. This information can be accessed as required, and anonymously and confidentially, if desired. In addition, it is cost-effective and convenient and can be accessed without having to leave one’s home [8]. However, when there is no monitoring of the quality or reliability of Internet-based information, the resultant information can be questionable. Parents can also be overwhelmed with both the amount of information available as well as the presentation of case studies of children with more complex or advanced presentations of conditions similar to those of their child.

Through the use of smartphones and mobile devices, information searching via social media and search engines is now a daily occurrence for many people [1]. Additionally, social networks can be established with other parents. Nationally and internationally based Facebook groups or pages are now common for rare conditions [7,9]. Furthermore, parents are using the Internet to receive information and emotional support from Web-based groups of parents facing similar challenges, while also critiquing care provision and health care policy [10]. However, information may not be presented in a balanced way via social networks. Thus, parents need to establish how reliable the information they are acquiring from Internet or social media resources is.

The aim of this study was to identify the Internet-sourced information and support content that meets the needs of parents of children with rare conditions for the purposes of developing an Irish website specifically designed to meet those needs. The objectives were to (1) ascertain parents’ general Internet usage patterns, (2) identify the nature of the information parents most frequently searched for, and (3) determine the effect the Internet-sourced information had on parents of children with rare conditions.

## Methods

This descriptive exploratory study used both qualitative and quantitative designs. Purposive nonprobability sampling was adopted where participant recruitment was facilitated by the Saoirse Foundation, which acted as the study’s gatekeeper. The study was advertised for 4 weeks on the gatekeeper’s website and in various linked voluntary organizations and social media links (Twitter, Facebook, blogs). The inclusion criterion was parents (18 years or older) of a child with a rare condition who used the Internet to source information about their child’s condition. A more detailed information leaflet was provided to the gatekeeper to distribute to parents who wished to know more about the study. Parents who wanted to participate in the study contacted the researcher and were scheduled to either (1) attend a focus group or (2) complete a questionnaire (Multimedia Appendix 1) that was open for 4 weeks.

The questionnaire was modeled on 2 previously published questionnaires by Porter and Edirippulige [11] and Tozzi et al [12], and 8 participants took part in the focus group to further refine the questionnaire. Written consent was sought prior to the commencement of the focus group. Once the focus group was completed, responses were fed back to the group, thus allowing participants verify their discussion, which was subsequently analyzed and categorized into themes (simple thematic analysis) [13].

The questionnaire consisted of 45 questions that were in the multiple-choice format apart from 2 open questions: (1) *the website(s) I most frequently visit is(are)* and (2) *if you were creating a website for parents of children with rare conditions, what is the ONE thing you would like to see on that website?* The resultant responses were analyzed by simple thematic analysis [13]. The questionnaire was divided into 4 sections, and although 128 respondents started the questionnaire, 93 respondents completed the questionnaire in full. Section 1 contained questions to confirm that the respondent was a parent of a child with a rare condition and used the Internet to source information about their child’s condition (inclusion criterion). A total of 7 respondents did not meet this inclusion criterion and were disqualified. So, the remaining respondents were only parents (Table 1).

**Table 1:** Overview of the questionnaire.

Section number	Section title	Number of questions	Number of respondents who commenced the section
1	Sources of information about your child’s condition	3	128 (7 disqualified, as they did not fulfill the inclusion criterion)
2	Information about your use of the Internet to find information about your child’s condition	23	121
3	Information about your child or children	8 (per child)	96
4	Information about yourself	11	93
	Total	45	93

Although participants had the option of completing Web- or paper-based versions of the questionnaire, all chose the Web-based option on SurveyMonkey (SurveyMonkey Inc). Ethical approval was obtained from the School of Nursing and Midwifery’s Research Ethics Committee, Trinity College Dublin, Ireland.

## Results

### Demographical Details

In Section 4 of the questionnaire, 93 respondents offered information about themselves (Table 2). The majority were mothers (87%, 81/93), aged between 35 and 49 years (67%, 62/93), living in urban areas (74%, 69/93) mostly in the Republic of Ireland (84%, 78/93). Half of the respondents (50%, 46/93) had a University degree, and 43% (40/93) were their child’s main carer (Table 2). Respondents who were not employed full time (84%, 78/93) were asked whether they left full-time employment to care for their child. Of these, 65% (51/78) selected *yes*, and the remaining 35% (27/78) selected either *no* or *not applicable*.

**Table 2:** Parents’ demographics (n=93).

Demographics		n (%)
**Gender**		
	Females	81 (87)
	Males	12 (13)
**Age (in years)**		
	18-34	22 (24)
	35-49	62 (67)
	50-64	9 (10)
**Country of residence**		
	Republic of Ireland	78 (84)
	Northern Ireland	6 (7)
	United Kingdom	7 (8)
	United States	2 (2)
**Area of residence**		
	City	20 (22)
	Town	30 (32)
	Village	19 (20)
	Rural	24 (26)
**Highest level of education**		
	Secondary school	23 (25)
	Vocational training	24 (26)
	University: Undergraduate degree	25 (27)
	University: Postgraduate degree	21 (23)
**Employment status**		
	Employed full time	15 (16)
	Employed part time	20 (22)
	Self-employed	3 (3)
	Your child or children’s main carer	40 (43)
	Homemaker	12 (13)
	Student	1 (1)
	Unemployed	2 (2)

In total, 117 children were reported on by their parents (Table 3). Furthermore, 78 parents reported on 1 child, 14 parents reported on 2 children, 1 parent reported on 3 children, and 2 parents reported on 4 children. Over half (54.7%, 64/117) the children were male and ranged in age from less than 12 months to 39 years, with the 4- to 7-year-old category being the largest (28.2%, 33/117). Ninety per cent (105/117) of children had a diagnosis, and 65.7% (69/105) received a diagnosis in their first 3 years of life. The majority (78.6%, 92/117) of children had a disability, and of these, a little more than half (53%, 49/92) had a physical and intellectual disability, 30% (28/92) had a physical disability, 10% (9/92) had an intellectual disability, and there was an equal spread (3%, 3/92) across sensory and neurodevelopmental disabilities, respectively. Some children (39.3%, 46/117) did not need assistive equipment. The remaining 71 children mainly needed equipment for moving (41.0%, 48/117), eating (19.7%, 23/117), and breathing (11.1%, 13/117; Table 3).

**Table 3:** Children’s demographics.

Demographics			n (%)
**Gender (n=117)**			
		Female	53 (45.3)
		Male	64 (54.7)
**Age (n=117)**			
		Under 12 months	5 (4.3)
		1-3	24 (20.5)
		4-7	33 (28.2)
		8-12	28 (23.9)
		13-19	15 (12.8)
		20-29	9 (7.7)
		30-39	3 (2.6)
**Does child have a diagnosis (n=117)**			
		No	12 (10.3)
		Yes	105 (89.7)
	**If yes, age at diagnosis (n=105)**		
		Under 12 months	36 (34.3)
		1-3	33 (31.4)
		4-7	16 (15.2)
		8-12	15 (14.3)
		13-19	4 (3.8)
		20-29	1 (0.9)
**Does the child's condition include a disability (n=117)**				
		No	25 (21.4)
		Yes	92 (78.6)
	**If yes, what is the nature of the disability (n=92)**		
		Physical and intellectual	49 (53)
		Physical	28 (30)
		Intellectual	9 (10)
		Sensory	3 (3)
		Neurodevelopmental	3 (3)
**Does your child use equipment for^a^** **(n=117)**				
		None required	46 (39.3)
		Moving	48 (41.0)
		Eating	23 (19.7)
		Breathing	13 (11.1)
		Speech	10 (8.6)
		Sitting	7 (5.9)
		Hearing	6 (5.1)
		Bathing; sleeping	3 (2.6)
		Writing; nonverbal; seeing	2 (1.7)
		Sensory integration	1 (0.9)

^a^Participants could choose as many options as applied to them.

### Internet Use

Respondents’ Internet usage is summarized in Table 4. The Internet was used predominantly at home (92.9%, 106/114), between 7 pm to midnight (42.9%, 49/114) and searched on a weekly basis (50.0%, 57/114; either *daily*, *several times,* or *once per week*) mostly on a PC or Mac (41.4%, 46/111). When searching the Internet for information on their child’s condition, 45.6% (52/114) *sometimes* found relevant information whereas 37.7% (43/114) found relevant information *most of the time*. All respondents (n=112) had an email address and 94.6% (106/112) had a Facebook account. Most (93.1%, 95/102) used general search engines (Google, Yahoo, Bing), and 28.4% (29/102) accessed specialized sites (Table 4). A total of 75 named websites were accessed (Multimedia Appendix 2).

**Table 4:** Internet use.

Internet usage		n (%)
**From where do you most often access the Internet? (n=114)**		
	Home	106 (92.9)
	Work	7 (6.1)
	Public library	0 (0)
	Other (Smartphone)	1 (0.9)
**What time of the day do you MOST OFTEN use the Internet (n=114)**		
	Midnight to 6 am	2 (1.8)
	7 am to midday	7 (6.1)
	1 pm to 6 pm	10 (8.8)
	7 pm to midnight	49 (42.9)
	No pattern	46 (40.4)
**How often do you use the Internet to find information about your child’s condition? (n=114)**		
	Every day	23 (20.2)
	Once a week	9 (7.9)
	Several times a week	25 (21.9)
	Once a month	10 (8.8)
	Several times a month	13 (11.4)
	Every few months	25 (21.9)
		9 (7.9)
**What device do you use MOST OFTEN to access the Internet? (n=111)**		
	PC or Mac	46 (41.4)
	Smartphone	42 (37.8)
	Tablet (iPad or similar)	23 (20.7)
**When using the Internet, how often are you able to find the information you are looking for? (n=114)**		
	Always	2 (1.8)
	Most of the time	43 (37.7)
	Sometimes	52 (45.6)
	Hardly ever	15 (13.2)
	Never	2 (1.8)
**Which of the following applies to you?^a^** **(n=112)**		
	Email	112 (100.0)
	Facebook	106 (94.6)
	Twitter	48 (42.9)
	Skype	57 (50.9)
	LinkedIn	31 (27.7)
	MSN or Messenger	23 (20.5)
	Blog	12 (10.7)
	Health-related apps	9 (8.0)
**How do you locate information about your child’s condition?^a^** **(n=102)**		
	Search engines (Google, Yahoo, Bing, etc)	95 (93.1)
	Specialized websites	29 (28.4)
	Orphanet	7 (6.9)
	Social media	7 (6.9)
	Recommendations from others	4 (3.9)

^a^Participants could choose as many options as applied to them.

Respondents were asked to rank which factors they took into account when choosing a website and could choose as many factors as applied to them. Of the 112 respondents who replied to this question, 65.2% (73/112) respondents ranked *relevant and accurate* as being the most important factor, closely followed by *trustworthiness* (62.5%, 70/112) and the website being *up to date* (60.7%, 68/112). In order of ranking, other factors included the website being *recommended to me by a health professional* (46.4%, 52/112), *easy to understand* (37.5%, 42/112), has *other websites linked within it* (8.0%, 9/112), and has a *nice layout* (5.4%, 6/112).

Respondents (n=101) ranked which information they searched for (1) when their child was first diagnosed or when they first had a concern about their child’s health and (2) what they search for at present (Table 5).

**Table 5:** Information topics sought by parents of children with rare conditions on the Internet (n=101; Participants who answered the questions could choose as many options as applied to them)

Rank	n^a^	Information searched for when first diagnosed	Change in rank	Rank	n^a^	Information searched for now
1	87	My child’s condition or symptoms	↔	1	71	My child’s condition or symptoms
2	77	My child’s diagnosis	↓2	2	70	The management of my child’s condition
3	69	The management of my child’s condition	↑1	3	51	The care of my child’s condition Treatments
4	66	Treatments Support groups	↑1 ↓1	4	47	My child’s diagnosis
5	64	Genetics	↓3	5	43	Support groups
6	57	The care of my child’s condition Child development	↑3 ↔	6	42	Child development
7	46	Organizations and societies	↓2	7	36	Research and innovation
8	40	Medical or health care professionals	↓2	8	35	Genetics
9	33	Research and innovation	↑2	9	33	Educational options Organizations and or societies
10	31	Early intervention options	↓7	10	30	Medical or health care professionals
11	30	Physical activities	↓1	11	29	Preventing complications Upcoming events or workshops
12	29	Hospitals, hospices, medical centers	↓3	12	28	Physical activities
13	28	Medicines or alternative treatments or therapies Nutrition	↓1 ↔	13	27	Nutrition
14	27	Preventing complications	↑3	14	22	Medicines or alternative treatments or therapies Financial assistance
15	26	Future pregnancies	↓2	15	21	Hospitals, hospices, medical centers State services
16	25	Educational options	↑7	16	17	Managing family dynamics
17	24	State services Financial assistance	↑2 ↑3	17	12	Early intervention options Future pregnancies
18	16	Where to get a second opinion	↓2	18	11	Accessing medicines or alternative treatments or therapies on the Internet
19	15	Upcoming events or workshops	↑8	19	8	Vaccinations
20	12	Managing family dynamics	↑4	20	7	Where to get a second opinion
21	11	Accessing medicines or alternative treatments or therapies on the Internet	↑3			
22	10	Vaccinations	↑3			

^a^Represents the number of participants who chose each information topic.

Overall there was not great variation in the ranking of information searched for over the 2 timelines. The first 10 ranked information topics underwent some minor reordering but remained in the top 10 across both timelines (apart from *early intervention options*), and *my child’s condition or symptoms* remained the highest ranked. *Early interventions options* ranked 10 when first diagnosed but decreased in ranking to 17 at the later time point.

Other topics increased in the rankings, for example, *managing family dynamics* ranked at 20 at the time of diagnosis compared with 16 at present. The greatest difference in ranking (7) was for *educational options,* which ranked 16 at time of diagnosis, but at present ranked 9. Similarly, *upcoming events or workshops* increased in ranking by 8 from 19 at the time of diagnosis to 11 at present. Interestingly *future pregnancies* changed little across timelines, ranking 15 at the time of diagnosis versus 17 at present**.**

### Impact

Having considered respondents’ usage of the Internet, next the respondents were asked whether Internet-sourced information influenced decisions they made about their child’s condition, and 98 of them replied. Most respondents considered that Internet-sourced information had some degree of influence, be it a *minor* or *some influence* (67%, 66/98) or a *major influence* (20%, 20/98), whereas for 10% (10/98), it had *no influence* and 2% (2/98) *did not know*.

Parents were asked about the impact on them of information found on the Internet by choosing relevant statements from a list, and they could choose as many as applied to them. In total, 86 parents replied to this question, and their choices are ranked in the order of popularity in Table 6. For 72% (62/86) of respondents, Internet-sourced information improved their understanding of their child’s condition. The next 2 highly ranked options were *enabled me to explain my child’s condition* (58%, 50/86) and *improved my ability to manage and care for my child’s condition* (57%, 49/86). For approximately a third of respondents (33%, 28/86), the impact of Internet-sourced information had served to increase their anxiety, while for 16% (14/86), it decreased their anxiety.

**Table 6:** The impact of Internet-sourced information on parents (n=86; Participants could choose as many options as applied to them)

Rank	Impact	n (%)
1	Improved my understanding of my child’s condition	62 (72)
2	Enabled me to explain my child’s condition	50 (58)
3	Improved my ability to manage and care for my child’s condition	49 (57)
4	Increased my anxiety	28 (33)
5	Was useful for diagnosing my child’s condition	20 (23)
6	Decreased my anxiety Was useful for accessing medicines or alternative treatments or therapies on the Internet	14 (16)
7	Made me change my medical or health care professional	8 (9)
8	Made me change my child’s food habits	6 (7)
9	Made me change my child’s physical activity	5 (6)
10	Was not useful Not sure	4 (5)
11	Empowered me	2 (2)
12	Helped me educate my health care professional Useful for making new contacts	1 (1)

### Parents Disseminating Internet-Sourced Information

Respondents used the Internet to communicate and link with others, for example, via email, Facebook, Twitter, and so forth (Table 4). More specifically, the respondents were asked whether they were registered in a Web-based forum or a social network group dedicated to their child’s condition, and of the 112 who answered this question, 80.4% (90/112) were. Of these 90 respondents, 86% (77/90) shared information about their child’s condition with these Internet communities.

In the context of health care, the respondents were asked whether they told their doctor or health care professional about the information they found on the Internet regarding their child’s condition. Seventy-six (78%, 76/97) who answered this question had relayed Internet-sourced information about their child, but there was variability regarding the degree of interest the doctor or health care professional had in the information. Of the 76 respondents, 50% (38/76) felt that their doctor or health care professional was *somewhat interested* in what they had found, whereas 22% (17/76) felt they were *very interested*, 16% (12/76) felt they were *not too interested*, 9% (7/76) felt that their doctor or health care professional was *not at all interested*, and 3% (2/76) *did not know*.

## Discussion

The 3 objectives of this study were to (1) ascertain parents general Internet usage patterns, (2) identify the nature of the information parents most frequently searched for, and (3) determine the impact Internet-sourced information had on parents of children with rare conditions. All objectives were met and are discussed in the following sections.

### Internet Use

All respondents were frequent and competent users of information and communication technology; all of them had an email account, and most (94.6%, 106/112) had a Facebook account. Respondents mostly searched the Internet from home, late in the evening, and were practiced at finding the information they sought; these patterns of general Internet usage concurred with similar literature [14,15].

This study found that parents were discerning when searching the Internet by comparing the content they found with their own experiences and knowledge. They took many factors into account such as the relevancy and accuracy of the information, how trustworthy and up to date it was, and who was disseminating the information; these judicious parent characteristics have been reported by others [14,16-18].

The parents in this study were well educated, and 90% of their children had a specific diagnosis. Despite this, respondents still accessed numerous sites seeking information regarding their child’s condition, prognosis, and management and to ask questions, a finding supported by Roche and Skinner [19] and others [8,16,20-22].

This study sought data on respondents’ information needs at 2 stages: at the time of their child’s diagnosis and at present. Although there was some variation across the 2 timelines significantly, the highest ranked item remained *my child’s condition or symptoms*. Similarly, there was consistency regarding the top 5 ranked information needs at the 2 timelines (Table 5). Pelentsov et al [23] have confirmed that despite the diverse characteristics of rare conditions, there is consistency regarding the common unmet needs of parents of children with rare conditions.

### Impact

The most significant impact of Internet-sourced information was the empowering effect it had on parents, particularly their improved understanding of their child’s condition. Internet-sourced information enabled them to explain their child’s condition and improved their ability to manage and care for their child. Some studies reported that Internet-sourced information resulted in parents changing their health care professional [19,24,25], which was also the case for 9% parents in this study (Table 6).

### Parents Disseminating Internet-Sourced Information

In this study, parents not only engaged with and consumed Internet-sourced information for their own needs, but they were active disseminators of newly sourced information about their child’s condition to their health care professionals and wider support network. Social media, Facebook in particular, provided instant access to other parents of children with rare conditions. In this study, the majority (86%) of those registered in a relevant Web-based forum or network shared information about their child’s condition. Traditionally peer support has been in the form of mailing lists, newsletters, discussion fora, and chat rooms [26]. However, it was evident in this study that Web-based engagement and exchange of information with other parents provided support and created a sense of belonging, which in turn reduced the feeling of exclusion, as has been reported by others [3,7,10,17,19,20,27-29].

Respondents were likely to talk to their health care professional about information they uncovered on the Internet, although not all health care professionals were interested in their findings. Few considered that the information had helped educate their health care professional, which is reported by others [30,31]. These findings were somewhat surprising, as even in 2002 doctors were being advised to be prepared to ask parents about their information needs and to discuss Internet use with them [32]. This lack of health care professional-parent exchange to determine what information parents are currently seeking and uncovering might be a missed opportunity in health care consultations and possibly improved child outcomes whereby health care professionals might better determine what services or supports parents require.

### Implications

Implications for health care practice include the impact Internet-sourced information is having on parent-health care professional dynamics. These include how information is shared, health care professionals’ interaction with parents when parents share with them the information they have sourced, and the subsequent changing nature of parents’ management of their child’s condition.

In this study, parents of children with rare conditions identified a number of key factors that should be considered when developing an Irish website. These include ensuring the following:

- The content is relevant, accurate, trustworthy, and up to date.
- The topics most frequently searched for (Table 5) are addressed.
- It contains a Web-based forum or a social network component.
- The website is integrated with social media and is mobile friendly.

These are fundamental factors considering that Internet-sourced information directly influences and impacts on parents (Table 6) and parents disseminate information widely to their health care professionals and to other parents and relevant networks.

### Limitations

As this was an Internet survey and participation was voluntary, it was not representative of all parents of children with rare conditions and most likely attracted participation from parents who were competent Internet users. The questionnaire consisted of 45 questions, and parents who had more than 1 child with a rare condition were asked to complete another 8 questions per additional child (Table 1). The length of the questionnaire might have contributed to the attrition of respondents as they progressed through the questionnaire. As the questionnaire was available on the Internet, it was accessed and completed by parents residing outside of Ireland (Table 2). Parents were asked which Internet-sourced information they sought during 2 phases in their child’s life: at the time of diagnosis and at present. However, this question was posed at 1 timepoint (at present) and parents were asked to retrospectively recall what information they were seeking when their child was first diagnosed. Nonetheless, this study did provide an insight into what parents used the Internet for and its impact. Further research into the relevance of information available to parents of children with rare conditions, the impact of Internet-sourced information on parents, the health care professionals’ response to parents, and the use of social media in parent-to-parent support is recommended.

### Conclusions

The findings of this study add to the body of emerging research that gives an insight into the use of, and reason for using, the Internet and information and communications technology by parents of children with rare conditions and the ever evolving parent-to-parent support systems (social and intellectual) via social media. What appears evident is that Internet and social media engagement facilitates the emergence of parents who are better informed and empowered, have greater understanding of the management and care of rare conditions, and are increasingly considered experts in their child’s care, specifically in how the particular condition is developing in their child [6]; many parents come prepared to health consultations with information sourced from the Internet [1].

## Appendix

Multimedia Appendix 1

Multimedia Appendix 2

## References

[1] Fergie G, Hilton S, Hunt K (2015). Young adults' experiences of seeking online information about diabetes and mental health in the age of social media. Health Expect.

[2] van den Bree MB, Miller G, Mansell E, Thapar A, Flinter F, Owen MJ (2013). The internet is parents' main source of information about psychiatric manifestations of 22q11.2 deletion syndrome (22q11.2DS). Eur J Med Genet.

[3] Pelentsov LJ, Laws TA, Esterman AJ (2015). The supportive care needs of parents caring for a child with a rare disease: a scoping review. Disabil Health J.

[4] Dragusin R, Petcu P, Lioma C, Larsen B, Jørgensen HL, Cox IJ, Hansen LK, Ingwersen P, Winther O (2013). FindZebra: a search engine for rare diseases. Int J Med Inform.

[5] Powell J, Inglis N, Ronnie J, Large S (2011). The characteristics and motivations of online health information seekers: cross-sectional survey and qualitative interview study. J Med Internet Res.

[6] Nicholl H, Doyle C, Begley T, Murphy M, Lawlor A, Malone H (2014). Developing an information leaflet on 22q11.2 deletion syndrome for parents to use with professionals during healthcare encounters. J Spec Pediatr Nurs.

[7] Anderson RP, Elliott EJ, Zurynski YA (2013). Australian families living with rare disease: experiences of diagnosis, health services use and needs for psychosocial support. Orphanet J Rare Dis.

[8] Nieuwboer CC, Fukkink RG, Hermanns JM (2013). Peer and professional parenting support on the Internet: a systematic review. Cyberpsychol Behav Soc Netw.

[9] Binford Hopf RB, Le Grange D, Moessner M, Bauer S (2013). Internet-based chat support groups for parents in family-based treatment for adolescent eating disorders: a pilot study. Eur Eat Disord Rev.

[10] Paterson BL, Brewer J, Stamler LL (2013). Engagement of parents in on-line social support interventions. J Pediatr Nurs.

[11] Porter A, Edirippulige S (2007). Parents of deaf children seeking hearing loss-related information on the internet: the Australian experience. J Deaf Stud Deaf Educ.

[12] Tozzi AE, Mingarelli R, Agricola E, Gonfiantini M, Pandolfi E, Carloni E, Gesualdo F, Dallapiccola B (2013). The internet user profile of Italian families of patients with rare diseases: a web survey. Orphanet J Rare Dis.

[13] Massey OT (2011). A proposed model for the analysis and interpretation of focus groups in evaluation research. Eval Program Plann.

[14] Hand F, McDowell DT, Mc Dowell DT, Glynn RW, Rowley H, Mortell A (2013). Patterns of internet use by parents of children attending a pediatric surgical service. Pediatr Surg Int.

[15] Rudi J, Dworkin J, Walker S, Doty J (2014). Parents' use of information and communications technologies for family communication: differences by age of children. Inf Commun Soc.

[16] Oprescu F, Campo S, Lowe J, Andsager J, Morcuende JA (2013). Online information exchanges for parents of children with a rare health condition: key findings from an online support community. J Med Internet Res.

[17] Glenn AD (2015). Using online health communication to manage chronic sorrow: mothers of children with rare diseases speak. J Pediatr Nurs.

[18] Ladd D (2015). General and health information challenges of patients with rare diseases: the importance of health information provision and web sites for locating rare disease resources. J Hosp Librariansh.

[19] Roche MI, Skinner D (2009). How parents search, interpret, and evaluate genetic information obtained from the internet. J Genet Couns.

[20] Kingsnorth S, Gall C, Beayni S, Rigby P (2011). Parents as transition experts? Qualitative findings from a pilot parent-led peer support group. Child Care Health Dev.

[21] Niela-Vilén H, Axelin A, Salanterä S, Melender HL (2014). Internet-based peer support for parents: a systematic integrative review. Int J Nurs Stud.

[22] Nölke L, Mensing M, Krämer A, Hornberg C (2015). Sociodemographic and health-(care-)related characteristics of online health information seekers: a cross-sectional German study. BMC Public Health.

[23] Pelentsov LJ, Fielder AL, Laws TA, Esterman AJ (2016). The supportive care needs of parents with a child with a rare disease: results of an online survey. BMC Fam Pract.

[24] Leonard H, Slack-Smith L, Phillips T, Richardson S, D'Orsogna L, Mulroy S (2004). How can the Internet help parents of children with rare neurologic disorders?. J Child Neurol.

[25] Skinner D, Schaffer R (2006). Families and genetic diagnosis in the genomic and Internet age. Infants Young Child.

[26] Eysenbach G, Powell J, Kuss O, Sa ER (2002). Empirical studies assessing the quality of health information for consumers on the world wide web: a systematic review. J Am Med Assoc.

[27] McGarvey B, Hart C (2008). Rehab.

[28] Gundersen T (2011). 'One wants to know what a chromosome is': the internet as a coping resource when adjusting to life parenting a child with a rare genetic disorder. Sociol Health Illn.

[29] Hall JG (2013). The role of patient advocacy/parent support groups. S Afr Med J.

[30] Powell J, Inglis N, Ronnie J, Large S (2011). The characteristics and motivations of online health information seekers: cross-sectional survey and qualitative interview study. J Med Internet Res.

[31] Schieppati A, Henter JI, Daina E, Aperia A (2008). Why rare diseases are an important medical and social issue. The Lancet.

[32] Tuffrey C, Finlay F (2002). Use of the internet by parents of paediatric outpatients. Arch Dis Child.

