# Supplementary material for: Internet Use by Parents of Children With Rare Conditions: Findings From a Study on Parents’ Web Information Needs
Source: J Med Internet Res. 2017 Feb 28;19(2):e51. doi: 10.2196/jmir.5834 (PMC5350458; doi:10.2196/jmir.5834)
Supplement: Multimedia Appendix 1 [file jmir_v19i2e51_app1.pdf]

# The RD-WIFI Questionnaire

**SOURCES OF INFORMATION ABOUT YOUR CHILD'S CONDITION**

1. Who is the person in your family MOST likely to seek information about your child's condition?

€ Myself
€ Spouse or partner
€ Other (please specify)

2. Where do you get information about your child's condition?
*Please choose all that apply.*

€ Internet or websites
€ Health care provider
€ Early intervention service
€ Books or literature
€ Family and friends
€ Word of mouth
€ Media e.g. television and newspapers
€ Other (please specify)

3. Do you use the Internet to get information about your child's condition?

€ Yes *(Please go to Q4)*
€ No *(Many thanks for the information that you have provided above. You do not need to proceed any further. Please return this questionnaire to Dr. Honor Nicholl using the addressed envelope).*

Copyright © 2014 Dr Aileen Lynch All Rights Reserved

## INFORMATION ABOUT YOUR USE OF THE INTERNET TO FIND INFORMATION ABOUT YOUR CHILD'S CONDITION

4. How comfortable are you using the Internet?
- € Very comfortable
- € Somewhat comfortable
- € Comfortable
- € Somewhat uncomfortable
- € Very uncomfortable

5. From where do you most often access the Internet?
- € Home
- € Work
- € Public library
- € Don't know
- € Other (please specify)

6. What time of the day do you MOST OFTEN use the Internet?
- € Midnight to 6am
- € 7am to midday
- € 1pm to 6pm
- € 7pm to midnight
- € No pattern

7. How often do you use the Internet to find information about your child's condition?
- € Every day
- € Once a week
- € Several times a week
- € Once a month
- € Several times a month
- € Every few months
- € Don't know

8. When you go online to look for information about your child's condition, how often are you able to find the information you are looking for?
- € Always
- € Most of the time
- € Sometimes
- € Hardly ever
- € Never
- € Don't know

Copyright © 2014 Dr Aileen Lynch All Rights Reserved

9. How many websites do you usually visit or browse when looking for information about your child's condition?

€ 1
€ 2 to 3
€ 4 to 5
€ 6 to 9
€ 10 to 20
€ More than 20
€ Don't know

10. Which of the following factors do you take into account when choosing a website? *Please choose all that apply.*

€ Trustworthiness e.g. author, qualifications, IP address
€ Up-to-date
€ Relevant and accurate
€ Nice layout
€ Easy to understand
€ Recommended to me by a medical/healthcare professional
€ Has other website links within it
€ Other (please specify)

11. What device do you use MOST OFTEN to access the Internet?

€ PC or Mac
€ Smartphone
€ Tablet (iPad or similar)
€ Other (please specify)

12. Which of the following applies to you? *Please choose all that apply.*

€ I have an email address
€ I have a Facebook account
€ I have a Twitter account
€ I have a Skype account
€ I have a LinkedIn account
€ I have an MSN/Messenger account
€ I write or contribute to a blog
€ I use health-related apps for my smartphone/tablet (please specify)

Copyright © 2014 Dr Aileen Lynch All Rights Reserved

Copyright © 2014 Dr Aileen Lynch All Rights Reserved

13. Are you registered in a forum or social network group dedicated to your child's condition?

€ Yes (*Please go to Q14)*

€ No (*Please go to Q15)*

14. Do you share information about your child's condition with these communities?

€ Yes

€ No

15. How do you find websites about your child's condition?

*Please choose all that apply.*

€ By visiting search engines such as Google, Yahoo, Bing, Ask Jeeves, Aol, Baidu etc.

€ By visiting Orphanet

€ By visiting a website that specialises in rare conditions (please specify)

€ Other (please specify)

16. Do you visit websites recommended by:

*Please choose all that apply.*

€ A doctor or healthcare professional

€ A friend or family member

€ Parents of children with rare conditions

€ Not applicable

€ Other (please specify)

17. The website(s) I **MOST FREQUENTLY** visit is(are):

Copyright © 2014 Dr Aileen Lynch All Rights Reserved

18. When your child was **FIRST** diagnosed or when you **FIRST** had a concern that something was wrong, what topics of information did you look for on the Internet? *Please choose all that apply.*

€ my child's diagnosis
€ my child's condition or symptoms
€ the care of my child's condition
€ the management of my child's condition
€ child development
€ managing family dynamics
€ medical / healthcare professionals
€ where to get a second opinion
€ early intervention options
€ educational options
€ treatments
€ alternative treatments / therapies
€ preventing complications
€ nutrition
€ physical activities
€ vaccinations
€ hospitals, hospices, medical centres
€ genetics
€ future pregnancies
€ support groups
€ organisations and/or societies
€ upcoming events or workshops
€ state services
€ financial assistance
€ research and innovation
€ accessing medicines or alternative treatments / therapies online
€ other topics (please specify)

Copyright © 2014 Dr Aileen Lynch All Rights Reserved

19. I **CURRENTLY** use the Internet to look for information about:
*Please choose all that apply.*

€ my child's diagnosis
€ my child's condition or symptoms
€ the care of my child's condition
€ the management of my child's condition
€ child development
€ managing family dynamics
€ medical / healthcare professionals
€ where to get a second opinion
€ early intervention options
€ educational options
€ treatments
€ alternative treatments / therapies
€ preventing complications
€ nutrition
€ physical activities
€ vaccinations
€ hospitals, hospices, medical centres
€ genetics
€ future pregnancies
€ support groups
€ organisations and/or societies
€ upcoming events or workshops
€ state services
€ financial assistance
€ research and innovation
€ accessing medicines or alternative treatments / therapies online
€ other topics (please specify)

20. Would you say that the information you find on the Internet influences the decisions you make about your child's condition?
€ Major influence *(Please go to Q21)*
€ Minor influence *(Please go to Q21)*
€ Some influence *(Please go to Q21)*
€ No influence at all *(Please go to Q22)*
€ Don't know *(Please go to Q22)*

Copyright © 2014 Dr Aileen Lynch All Rights Reserved

21. The information I found on the Internet:
    *Please choose all that apply.*

€ Was not useful
€ Was useful for diagnosing my child's condition
€ Improved my understanding of my child's condition
€ Improved my ability to manage and care for my child's condition
€ Enabled me to explain my child's condition
€ Increased my anxiety
€ Decreased my anxiety
€ Made me change my medical / healthcare professional
€ Made me change my child's food habits
€ Made me change my child's physical activity
€ Was useful for accessing medicines or alternative treatments / therapies online
€ Not sure
€ Other (please specify)

22. Have you told your doctor or healthcare professional about the information you found on the Internet regarding your child's condition?

€ Yes *(Please go to Q23)*
€ No *(Please go to Q25)*
€ Don't know *(Please go to Q25)*

23. How did you tell your doctor or healthcare professional about the information you found on the Internet?
    *Please choose all that apply.*

€ I spoke to him/her directly
€ I used email
€ I used Facebook
€ I used other social networks
€ I used telemedicine services
€ Other (please specify)

24. When you told your doctor or healthcare professional about the information you found on the Internet, how interested where they?

€ Very interested
€ Somewhat interested
€ Not too interested
€ Not at all interested
€ Don't know

Copyright © 2014 Dr Aileen Lynch All Rights Reserved

25. How important is it that a website for parents of children with rare conditions is accessible to anyone with a disability?
- € Very important
- € Important
- € Moderately important
- € Of little importance
- € Unimportant
- € Don’t know

26. Should a website for parents of children with rare conditions be available in the Irish language?
- € Yes
- € No
- € Don’t know

## INFORMATION ABOUT YOUR CHILD OR CHILDREN

27. How many of your children have a rare condition?
- € 1
- € 2
- € 3
- € 4+

### CHILD 1

28. Child’s age
- € Under 12 months
- € 1 to 3
- € 4 to 7
- € 8 to 12
- € 13 to 19
- € 20 to 29
- € 30 to 39
- € 40 to 49
- € 50+

29. Child’s sex
- € Male
- € Female

30. Does your child have a diagnosis?
- € Yes *(Please go to Q31)*
- € No *(Please go to Q32)*

Copyright © 2014 Dr Aileen Lynch All Rights Reserved

31. Child's age when diagnosed
€ Under 12 months
€ 1 to 3
€ 4 to 7
€ 8 to 12
€ 13 to 19
€ 20 to 29
€ 30 to 39
€ 40 to 49
€ 50+

32. Does your child's condition include a disability?
€ Yes *(Please go to Q33)*
€ No *(Please go to Q34)*

33. If you answered Yes to question 32, please specify the type or types of disability:
€ Physical
€ Intellectual
€ Physical and intellectual
€ Other (please specify)

34. Does your child use equipment for:
*Please choose all that apply.*
€ Moving
€ Eating
€ Breathing
€ Hearing
€ Speech
€ None
€ Other (please specify)

**If you have additional children with a rare condition please complete the following pages for each child.**

**Otherwise please go to page 14.**

Copyright © 2014 Dr Aileen Lynch All Rights Reserved

Copyright © 2014 Dr Aileen Lynch All Rights Reserved

**CHILD 2**

35. Child’s age
- € Under 12 months
- € 1 to 3
- € 4 to 7
- € 8 to 12
- € 13 to 19
- € 20 to 29
- € 30 to 39
- € 40 to 49
- € 50+

36. Child’s sex
- € Male
- € Female

37. Does your child have a diagnosis?
- € Yes *(Please go to Q38)*
- € No *(Please go to Q39)*

38. Child’s age when diagnosed
- € Under 12 months
- € 1 to 3
- € 4 to 7
- € 8 to 12
- € 13 to 19
- € 20 to 29
- € 30 to 39
- € 40 to 49
- € 50+

39. Does your child’s condition include a disability?
- € Yes *(Please go to Q40)*
- € No *(Please go to Q41)*

40. If you answered Yes to question 39, please specify the type or types of disability:
- € Physical
- € Intellectual
- € Physical and intellectual
- € Other (please specify)

Copyright © 2014 Dr Aileen Lynch All Rights Reserved

41. Does your child use equipment for:
*Please choose all that apply.*
€ Moving
€ Eating
€ Breathing
€ Hearing
€ Speech
€ None
€ Other (please specify)

**CHILD 3**

42. Child’s age
€ Under 12 months
€ 1 to 3
€ 4 to 7
€ 8 to 12
€ 13 to 19
€ 20 to 29
€ 30 to 39
€ 40 to 49
€ 50+

43. Child’s sex
€ Male
€ Female

44. Does your child have a diagnosis?
€ Yes *(Please go to Q45)*
€ No *(Please go to Q46)*

45. Child’s age when diagnosed
€ Under 12 months
€ 1 to 3
€ 4 to 7
€ 8 to 12
€ 13 to 19
€ 20 to 29
€ 30 to 39
€ 40 to 49
€ 50+

Copyright © 2014 Dr Aileen Lynch All Rights Reserved

46. Does your child's condition include a disability?
€ Yes *(Please go to Q47)*
€ No *(Please go to Q48)*

47. If you answered Yes to question 46, please specify the type or types of disability:
€ Physical
€ Intellectual
€ Physical and intellectual
€ Other (please specify)

48. Does your child use equipment for:
*Please choose all that apply.*
€ Moving
€ Eating
€ Breathing
€ Hearing
€ Speech
€ None
€ Other (please specify)

**CHILD 4**

49. Child's age
€ Under 12 months
€ 1 to 3
€ 4 to 7
€ 8 to 12
€ 13 to 19
€ 20 to 29
€ 30 to 39
€ 40 to 49
€ 50+

50. Child's sex
€ Male
€ Female

Copyright © 2014 Dr Aileen Lynch All Rights Reserved

51. Does your child have a diagnosis?
€ Yes *(Please go to Q52)*
€ No *(Please go to Q53)*

52. Child's age when diagnosed
€ Under 12 months
€ 1 to 3
€ 4 to 7
€ 8 to 12
€ 13 to 19
€ 20 to 29
€ 30 to 39
€ 40 to 49
€ 50+

53. Does your child's condition include a disability?
€ Yes *(Please go to Q54)*
€ No *(Please go to Q55)*

54. If you answered Yes to question 53, please specify the type or types of disability:
€ Physical
€ Intellectual
€ Physical and intellectual
€ Other (please specify)

55. Does your child use equipment for:
*Please choose all that apply.*
€ Moving
€ Eating
€ Breathing
€ Hearing
€ Speech
€ None
€ Other (please specify)

Copyright © 2014 Dr Aileen Lynch All Rights Reserved

## INFORMATION ABOUT YOURSELF

56. Are you the child or children's:
€ Father
€ Mother
€ Legal guardian
€ None of the above

57. Are you?
€ Male
€ Female

58. What is your age?
€ Under 18
€ 18 to 34
€ 35 to 49
€ 50 to 64
€ 65 to 79
€ 80 or older

59. Do you live in?
€ the Republic of Ireland
€ Northern Ireland
€ the United Kingdom
€ Europe
€ Other (please specify)

60. What location BEST describes where you live?
€ City
€ Town
€ Village
€ Rural

61. What is your HIGHEST level of education?
€ Primary school
€ Secondary school
€ Vocational training
€ Undergraduate degree

Copyright © 2014 Dr Aileen Lynch All Rights Reserved

€ Postgraduate degree

62. Are you:

€ Employed full-time *(Please go to Q64)*
€ Employed part-time *(Please go to Q63)*
€ Self-employed *(Please go to Q63)*
€ Your child/children’s main carer *(Please go to Q63)*
€ A homemaker *(Please go to Q63)*
€ A student *(Please go to Q63)*
€ Unemployed *(Please go to Q63)*
€ Other (please specify) *(Please go to Q63)*

63. If not employed full-time, did you leave your full-time job to care for your child/children?

€ Yes
€ No
€ Not applicable

64. How comfortable are you SPEAKING English?

€ Very comfortable
€ Somewhat comfortable
€ Comfortable
€ Somewhat uncomfortable
€ Very uncomfortable

65. How comfortable are you READING English?

€ Very comfortable
€ Somewhat comfortable
€ Comfortable
€ Somewhat uncomfortable
€ Very uncomfortable

66. Finally, if you were creating a website for parents of children with rare conditions, what is the **ONE** thing you would like to see on that website?

**Thank you for your time, input to and support of this study.**

Copyright © 2014 Dr Aileen Lynch All Rights Reserved

References: Porter & Edirippulige (2007), Roberts (2010) and Tozzi *et al.* (2013).

Copyright © 2014 Dr Aileen Lynch All Rights Reserved
