# Supplementary material for: Internet Use by Parents of Children With Rare Conditions: Findings From a Study on Parents’ Web Information Needs
Source: J Med Internet Res. 2017 Feb 28;19(2):e51. doi: 10.2196/jmir.5834 (PMC5350458; doi:10.2196/jmir.5834)
Supplement: Multimedia Appendix 2 [file jmir_v19i2e51_app2.pdf]

Website Name	Website Address	Number of times mentioned
22Q Foundation	www.22q.org	2
22Q11 Charity Ireland	www.22q11ireland.org	2
Aaron's Ohtahara	www.ohtahara.org	1
Aicardi Syndrome	www.aicardisyndrome.org	1
Allergy Ireland	www.allergy-ireland.ie	1
Anaphylaxis Ireland	www.irishanaphylaxis.org	1
Autoinflammatory Alliance	www.nomidalliance.org	1
Boston University Medical Campus, Boston University	www.bumc.bu.edu	1
British Medical Journal	www.bmj.com	1
CDKL5 UK	www.cdkl5uk.org	1
Child Growth Foundation	www.childgrowthfoundation.org	1
Child Lung Foundation: ChILD	www.childlungfoundation.org	1
Children's Craniofacial Association	www.ccakids.com	1
Chromosome 18	www.chromosome18.org	1
Cohen Syndrome	www.cohen-syndrome.org	1
DBA UK	www.diamondblackfan.org.uk	1
Disability is Natural	www.disabilityisnatural.com	1
Epilepsy Ireland	www.epilepsy.ie	3
Epilepsy.com	www.epilepsy.com	1
EURORDIS	www.eurordis.org	1
Facebook (including support groups and organisations)	www.facebook.com	22
Foundation for Mitochondrial Medicine	www.mitochondrialdiseases.org	1
Fragile X Ireland	www.fragilexireland.org	1
Genetic and Rare Disorders Organisation (GRDO)	www.grdo.ie	1
Genetic.Org	www.genetic.org	1
Google	www.google.com	7
Great Ormond Street Hospital Children's Charity	www.gosh.org	1
Histiocytic Disorders	www.histio.org	1
Hunter Syndrome Foundation	www.huntersyndromefoundation.org	1
Hypermobility Syndromes Association	www.hypermobility.org	1
Inclusion Ireland	www.inclusionireland.ie	1
International Children's Palliative Care Network	www.icpcn.org	1
International Foundation for CDKL5 Research	www.Cdkl5.com	1
International Patient Organisation for C1 Inhibitor Deficiencies	www.haei.org	1
Irish Autism Action	www.autismireland.ie	1
Kids With Food Allergies	www.kidswithfoodallergies.org	1
Klinefelter's Syndrome UK	www.ksa-uk.co.uk	1
LGS Foundation: Lennox-Gastaut Syndrome	www.lgsfoundation.org	2
Liam's Lodge	www.liamslodge.com	1

Marfan Syndrome Support Group Ireland	www.marfan.ie	2
Max Appeal!	www.maxappeal.org.uk	1
Mayo Clinic	www.mayoclinic.org	1
Metachromatic Leukodystrophy Foundation	www.MLDfoundation.org	1
MitoAction	www.mitoaction.org	3
Monarch Initiative	www.monarchinitiative.org	1
Mumsnet	www.mumsnet.com	1
National Center for Learning Disabilities	www.ncld.org	1
National Fragile X Foundation	www.fragilex.org	3
National Marfan Foundation	www.marfan.org	2
National Organization for Rare Disorders	www.rarediseases.org	1
Neurofibromatosis Association of Ireland	www.nfaireland.ie	1
Newcastle Hospitals - Childrens Services	www.newcastle-hospitals.org.uk	1
NINDS Alpers' Disease Information Page	www.ninds.nih.gov/disorders/alpersdisease/alpersdisease.htm	1
NINDS Lissencephaly Information Page	www.ninds.nih.gov/disorders/lissencephaly/lissencephaly.htm	1
Orphanet	www.orpha.net	1
Osteogenesis Imperfecta Foundation	www.oif.org	5
Pachygyria - Right Diagnosis	www.rightdiagnosis.com/p/pachygyria/intro.htm	1
Rollercoaster	www.Rollercoaster.ie	1
Rubinstein-Taybi Syndrome	www.rubinstein-taybi.com	1
Special Needs Parents Association	www.specialneedsparents.ie	1
Spina Bifida Hydrocephalus Ireland	www.sbhi.ie	1
Stanford Medicine	www.med.stanford.edu	1
SWAN UK	www.undiagnosed.org.uk	1
Teeter's Page on Apert's syndrome	www.apert.org	1
The Brittle Bone Society	www.brittlebone.org	6
The Histiocytosis Research Trust	www.hrtrust.org	1
The MAGIC Foundation	www.magicfoundation.org	1
The Society for Mucopolysaccharide Diseases	www.mpssociety.co.uk	1
The United Mitochondrial Disease Foundation	www.umdf.org	4
The US Hereditary Angioedema Association	www.haea.org	1
Tuberous Sclerosis Alliance	www.tsalliance.org	1
Unique The Rare Chromosome Disorder Support Group	www.rarechromo.org	7
VCFS Educational Foundation	www.vcfsef.org	1
WebMD	www.webmd.com	1
XLH Network (X-Linked Hypophoshpatemia)	www.xlhnetwork.org	1

Supplementary Table 1. A list of the most frequently visited websites (Q17 in questionnaire).
